# Anti-Inflammatory Mesenchymal Stromal Cell-Derived Extracellular Vesicles Improve Pathology in Niemann–Pick Type C Disease

**DOI:** 10.3390/biomedicines9121864

**Published:** 2021-12-08

**Authors:** Lien Van Hoecke, Caroline Van Cauwenberghe, Verena Börger, Arnout Bruggeman, Jonas Castelein, Griet Van Imschoot, Elien Van Wonterghem, Robin Dittrich, Wouter Claeys, Junhua Xie, Bernd Giebel, Roosmarijn E. Vandenbroucke

**Affiliations:** 1 VIB Center for Inflammation Research, VIB, 9052 Ghent, Belgium; lien.vanhoecke@irc.vib-ugent.be (L.V.H.); caroline.vancauwenberghe@thomasmore.be (C.V.C.); Arnout.Bruggeman@irc.VIB-UGent.be (A.B.); jonas.castelein@irc.vib-ugent.be (J.C.); Griet.VanImschoot@irc.VIB-UGent.be (G.V.I.); Elien.VanWonterghem@irc.VIB-UGent.be (E.V.W.); Wouter.Claeys2@irc.vib-ugent.be (W.C.); Junhuax@irc.vib-ugent.be (J.X.); 2 Department of Biomedical Molecular Biology, Ghent University, 9000 Ghent, Belgium; 3 Institute for Transfusion Medicine, University Hospital Essen, University Duisburg-Essen, 45147 Essen, Germany; verena.boerger@uk-essen.de (V.B.); robin.dittrich@uk-essen.de (R.D.); bernd.giebel@uk-essen.de (B.G.); 4 Liver Research Center Ghent, Hepatology Research Unit, Department of Internal Medicine and Pediatrics, Ghent University, 9000 Ghent, Belgium

**Keywords:** Niemann–Pick type C disease, extracellular vesicles, mesenchymal stem cell

## Abstract

Niemann–Pick type C (NPC) disease is a rare neurovisceral lipid storage disease with progressive neurodegeneration, leading to premature death. The disease is caused by loss-of-function mutations either in the *NPC1* or *NPC2* gene which results in lipid accumulation in the late endosomes and lysosomes. The involved disease mechanisms are still incompletely understood, making the design of a rational treatment very difficult. Since the disease is characterized by peripheral inflammation and neuroinflammation and it is shown that extracellular vesicles (EVs) obtained from mesenchymal stromal cells (MSCs) provide immunomodulatory capacities, we tested the potential of MSC-EV preparations to alter NPC1 disease pathology. Here, we show that the administration of an MSC-EV preparation with in vitro and in vivo confirmed immune modulatory capabilities is able to reduce the inflammatory state of peripheral organs and different brain regions of NPC1-diseased mice almost to normal levels. Moreover, a reduction of foamy cells in different peripheral organs was observed upon MSC-EV treatment of NPC1^−/−^ mice. Lastly, the treatment was able to decrease microgliosis and astrogliosis, typical features of NPC1 patients that lead to neurodegeneration. Altogether, our results reveal the therapeutic potential of MSC-EVs as treatment for the genetic neurovisceral lipid storage disease NPC, thereby counteracting both central and peripheral features.

## 1. Introduction

Niemann–Pick disease type C (NPC) is a neurovisceral disorder represented by a dominant progressive neurodegenerative involvement in 90% of the patients [1]. It is a lipid storage disease characterized by oxidative stress and impaired trafficking of unesterified cholesterol and glycosphingolipids in the late endosome and lysosome system. The clinical symptoms associated with NPC are heterogeneous and vary from patient to patient [2]. None of the symptoms on its own are specific for the disease, it is rather the combination of different manifestations that is indicative for NPC [3]. In general, two types of symptoms can be distinguished, namely systemic and neurologic symptoms. The systemic symptoms include enlargement and inflammatory damage of liver and spleen and pulmonary diseases caused by the presence of foamy cells. Neuropathologically the disease is characterized by ballooned neurons distended with lipid storage, widespread neuronal loss, microgliosis, and astrogliosis [4]. Furthermore, neural stem cells derived from NPC disease models have decreased ability for self-renewal and neuronal differentiation. In a cross-sectional study of NPC patients, Walterfang et al. showed that neuroinflammation, particularly in white matter, substantiate structural and degenerative changes in the brain of NPC patients [5]. In addition to being a disrupted lysosome function disorder, NPC demonstrates a significantly dysregulation of innate immunity [6].

At present, there is no cure for NPC1 patients as the exact mechanism behind the disease is insufficiently known [6]. Therefore, the treatment of NPC1 is focused on the alleviation of symptoms like management of seizures with antiepileptic drugs, tremor might be improved in some cases by anticholinergic drugs, and spasticity may also be managed by physiotherapy [7]. Nevertheless, there is an urgent need for novel therapies that address the systemic as well as the neurodegenerative symptoms more efficient. As the disease is characterized by its proinflammatory status, immune modulatory treatments appear an attractive therapeutic strategy.

Mesenchymal stromal cells (MSCs) emerged as a potential NPC therapy [8]. These cells promote anti-inflammatory immune responses and suppress pro-inflammatory responses [9,10]. More precisely, they promote regulatory T-cell formation, shift proinflammatory macrophage functions towards anti-inflammatory activities, and secrete anti-inflammatory cytokines. Due to these immunomodulatory characteristics, MSCs represent a promising tool for cell therapy in different inflammatory disease settings. In one of the first studies, back in 2004, Le Blanc and colleagues successfully used bone marrow-derived MSCs to suppress graft-vs.-host disease (GvHD) symptoms in a severe treatment resistant grade IV acute patient [11]. Since then, MSCs were administered as immunomodulatory drug to GvHD patients and to patients suffering from other inflammatory diseases in many different studies. Amongst others, two clinical phase III studies were performed to treat steroid-refractory GvHD patients with commercial MSC products. While the first study on adult GvHD study failed to show efficacy [12,13], the second study on pediatric GvHD patient showed therapeutic MSC effects [14,15].

Increasing evidence indicates that MSCs exert their therapeutic effects in a paracrine manner rather than by direct cellular interactions as it was assumed for many years before [8,16,17,18,19,20]. It was shown that secreted, immune-modulating, nano-sized extracellular vesicles (EVs) mediate these paracrine effects. Preclinical data and first clinical data indicate that EVs, especially those derived from mesenchymal stem cells (MSCs), show great potential as therapeutic active agents in tissue regeneration and immune response modulation [21,22,23,24,25]. This is exemplified by an individual treatment attempt of an otherwise treatment resistant acute GvHD patient, who was treated over a 2-week period with escalating doses of an MSC-EV product. During the therapy, the aGvHD symptoms declined significantly, and the patient was stable for more than 4 months [26].

Here, we explored the potential of MSC-EVs, which were manufactured identically to those that were applied to the GvHD patient, in suppressing NPC associated symptomatology. We show that the intravenous administration of MSC-EVs into a mouse model for NPC1, namely Balb/cNctr-Npc1m1N/J mice, is able to reverse typical neurologic and systemic symptoms. More precisely, the MSC-EV treatment is able to lower inflammation in different brain regions and lower microgliosis and astrogliosis. Moreover, also a reduction in peripheral inflammation and number of foamy cells in the spleen, liver and lungs was observed upon MSC-EV treatment.

Taken together, this study demonstrates the potential of MSC-EVs in suppressing symptoms of the genetic neurovisceral lipid storage disease NPC. Administered MSC-EVs counteract both central and peripheral disease symptoms.

## 2. Results

### 2.1. MSC-EV Treatment Strategy of NPC1^+/+^ and NPC1^−/−^ Mice

**Figure 1:**
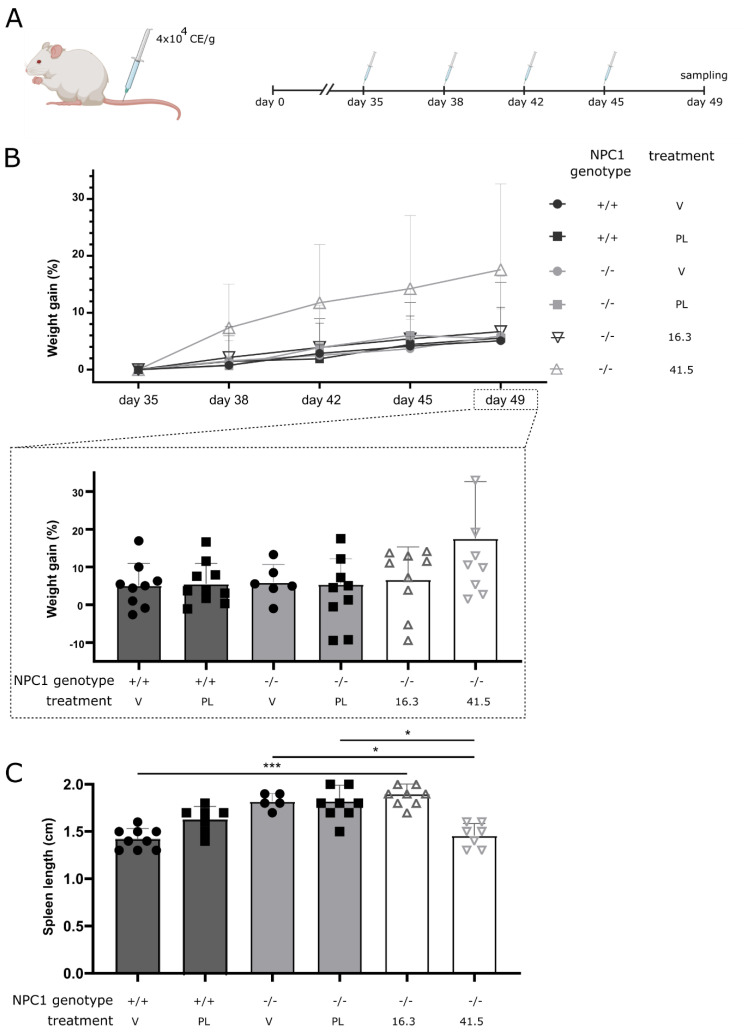
MSC41.5-EV administration restores overall weight gain and spleen size in NPC1^−/−^ mice. (**A**) NPC1^−/−^ mice and wild type littermates received 4 × 10^4^ cell equivalents/gram (CE/g) EVs by intravenous injection into lateral tail vein. EVs were diluted in 10 mM HEPES containing 0.9% NaCl buffer. Control mice were injected with vehicle (buffer only). Three different EV preparations were tested: platelet lysate-derived EVs (PL), MSC16.3-EVs (16.3) and MSC41.5-EVs (41.5) harvested from conditioned media of MSCs expanded from donor stocks MSC16.3 or MSC41.5, respectively. (**B**,**C**) At age of 7 weeks, weight gain (**B**) compared to start of treatment and area of spleen (**C**) were calculated. Data are shown as mean ± SEM (*n* = 7 to 9 mice per group). Statistical analyses on datasets were performed by Kruskal–Wallis test. Asterisks indicate statistical significance (* *p* < 0.05, *** *p* < 0.001). V: vehicle; PL: platelet derived EVs; 16.3: MSC16.3-EVs; 41.5: MSC41.5-EVs.

Here, we compared the therapeutic potential of two different MSC-EV preparations on NPC disease symptoms in NPC1^−/−^ mice that carry a spontaneous loss of function mutation within the *Npc1* gene (deletion of 11 out of its 13 transmembrane domains) [27,28,29]. Both MSC-EV preparations were produced according to a standardized procedure, i.e., MSCs were expanded in non-EV depleted human platelet lysate supplemented media wit−h media changing intervals of 48 h. EVs from conditioned media were prepared according to an optimized polyethylenglycol-based (PEG) precipitation method including a final ultracentrifugation polishing step [30,31]. Although both obtained MSC-EV preparations (MSC16.3-EV and MSC41.5-EV) revealed comparable EV characteristics, we previously reported that they exert different activities in an ischemic stroke as well as an optimized murine graft-versus.-host disease (GvHD) model and differed in their ability to modulate allogenic immune responses in vitro. Indeed, while the MSC41.5-EV preparation showed immunomodulatory activities in both animal models and in vitro, MSC16.3-EV failed to show therapeutic efficacy [32,33]. To compare their impact on typical systemic and neurologic features of the early-onset NPC1 pathology, aliquots of both MSC-EV preparations and of an EV preparation obtained from fresh, human platelet lysate supplemented MSC cultivation media (PL EV) were repetitively administered to NPC1^−/−^ mice. As displayed in Figure 1A, the treatment regime consisted of four intravenous injections between five and seven weeks of age. As negative control, mice were treated with the EV vehicle. At seven weeks of age, the effect of the treatment on both systemic and neurologic symptoms typical for NPC1 pathology was evaluated.

### 2.2. MSC41.5-EV but Not MSC16.3-EV Treatment Protects against NPC1^−/−^-Associated Weight Loss and Spleen Enlargement

Similar to infants with NPC1 disorder, also NPC1^−/−^ mice show a progressive lower weight compared to their wildtype littermates (Figure S1) [2]. To evaluate if this phenotype can be reversed by MSC-EV treatment, we followed up the weight of the treated and control mice over the treatment period and plotted their weight gain in comparison to the weight at the start of the treatment. All treatment groups except the MSC41.5-EV treated mice revealed a comparable weight gain over the treatment time. Indeed, NPC1^−/−^ mice that received MSC41.5-EVs gained, however not significantly, more weight over time compared with that of all other treatment groups (Figure 1B).

Next, we evaluated the effect on splenomegaly, another typical characteristic of NPC1 pathology (Figure 1C). MSC41.5-EVs, but not MSC16.3-EVs, were able to reduce the typical enlargement of the spleen observed in NPC1^−/−^ mice treated with vehicle or PL EVs. Thus, our data indicate that MSC-EV preparations with therapeutic features provide the potential to effectively suppress NPC symptoms. As before, however, we observed that despite comparable EV characteristics, not all MSC-EV preparations obtain the required activity.

### 2.3. MSC41.5-EV but Not MSC16.3-EV Treatment Reduces Peripheral Inflammation in NPC1^−/−^ Mice

**Figure 2:**
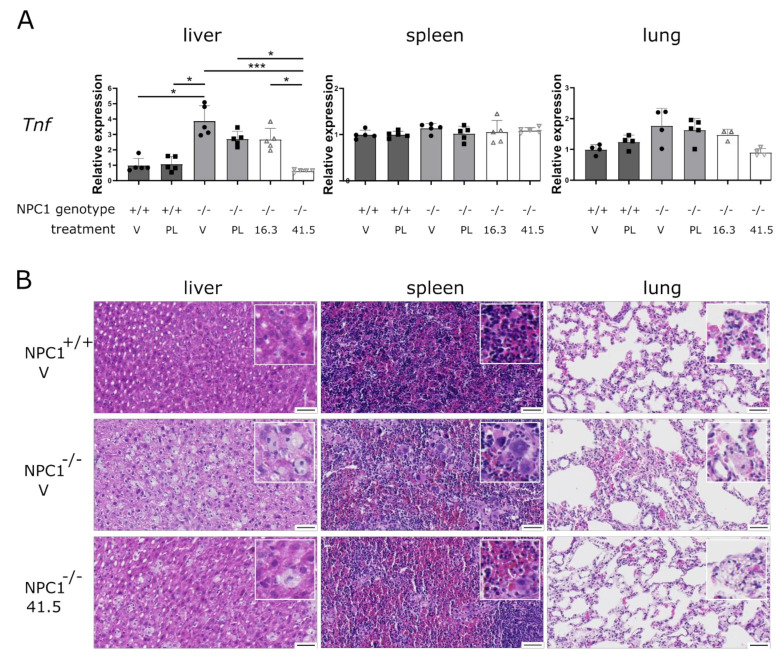
MSC41.5-EV treatment reduces NPC1^−/−^ pathology in peripheral organs. (**A**,**B**) NPC1^−/−^ mice and NPC1^+/+^ littermates are treated four times by intravenous injection into lateral tail vein with vehicle (V), platelet derived EVs (PL), EVs harvested from donor MSC16.3 (16.3) or donor MSC41.5 (41.5) conditioned media. At age of 7 weeks, liver, spleen, and lungs were isolated followed by Tnf expression analysis (**A**) and hematoxylin and eosin (H & E) staining (**B**). qRT-PCR results are represented relative to NPC1^+/+^ vehicle condition. Data are shown as mean ± SEM (*n* = 5 mice per group). Statistical analyses on datasets were performed by Kruskal–Wallis test. Asterisks indicate statistical significance (* *p* < 0.05, *** *p* < 0.001). Scale bars represent 50 µm.

As there is multiple evidence that tumor necrosis factor (TNF) plays a key role in the pathogenesis of NPC1 in peripheral organs [34,35,36,37,38,39,40,41], we investigated whether MSC41.5-EV treatment decreases the expression of this pro-inflammatory cytokine (Figure 2A). At the gene expression level, we observed that *Tnf* expression is significantly increased in the liver and showed an increased trend in the lungs of NPC1^−/−^ mice compared to that of wild type NPC1^+/+^ littermates. Following MSC41.5-EV treatment, but not after administration of MSC16.3-EVs or PL-EVs, *Tnf* expression is significantly suppressed to normal, wild type levels in the liver. The same trend can be observed in the lungs. In spleen, no *Tnf* upregulation nor suppression could be observed due to NPC1 deficiency and MSC-EV treatment, respectively.

To determine how MSC-EV treatment affects liver, spleen, and lung histology, H & E stained-tissue sections were examined. NPC1^−/−^ mice extensively accumulate cytoplasmic lipids, resulting in foam cell formation [42]. Such foam cells are also formed in the liver, spleen, and lung of our NPC1^−/−^ mice, but not in their NPC1^+/+^ littermates (Figure 2B). In contrast, we hardly detected foam cells in NPC1^−/−^ mice that were treated with MSC41.5-EVs. Neither treatment with PL- or MSC16.3-EVs was able to reduce the number of foam cells in spleen, liver, and lungs of NPC1^−/−^ mice (Figure S2).

Taken together, treatment of NPC1 pathology with EVs derived from conditioned media of the donor stock MSC41.5 but not MSC16.3 is able to reduce typical peripheral NPC1 symptoms.

### 2.4. MSC41.5-EV Treatment Reduces Inflammation in Different Brain Regions in NPC1^−/−^ Mice

**Figure 3:**
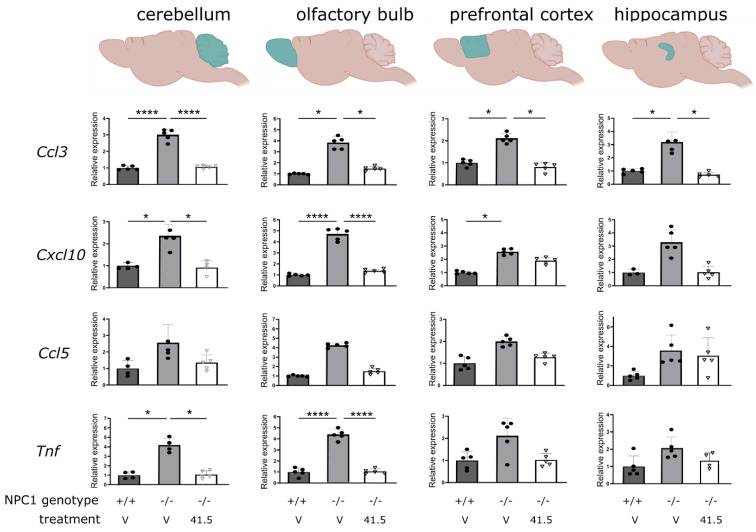
MSC41.5-EV treatment of NPC1^−/−^ mice reduces neuroinflammation in different brain regions. NPC1^−/−^ mice and wild type littermates are four times injected intravenous into lateral tail vein with vehicle or MSC-EVs derived from human MSC donor 41.2 (MSC-EV 41.2). At age of 7 weeks, different brain regions were isolated and *Ccl3*, *Cxcl10*, *Ccl5* and *Tnf* gene expression was analyzed. Results are represented relative to NPC1^+/+^ vehicle condition. Data are shown as mean ± SEM (*n* = 5 mice per group). Statistical analyses on datasets were performed by Kruskal–Wallis test. Asterisks indicate statistical significance (* *p* < 0.05, **** *p* < 0.0001).

In addition to the spectrum of visceral symptoms, NPC1 pathology also impacts the central nervous system (CNS) and leads to neuroinflammation and -degeneration [43]. Here, we explored the expression strength of several cytokines and chemokines in different brain regions (Figure 3). As expected, compared to their healthy littermates, a significant increase in the expression of almost all tested inflammatory genes was found in NPC1^−/−^ mice. Strikingly, administration of MSC41.5-EVs reduced this expression to almost normal levels and this holds true for all investigated brain regions, namely cerebellum, olfactory bulb, prefrontal cortex, and hippocampus. These results indicate that treatment with MSC41.5-EVs is able to suppress neuroinflammation in NPC1^−/−^ back to normal wild type level. In contrast, again neither treatment with PL EVs nor MSC16.3-EVs were able to reduce neuroinflammation (Figure S3).

### 2.5. MSC-EV Treatment Mitigates Microgliosis and Astrogliosis in NPC1^−/−^ Mice

**Figure 4:**
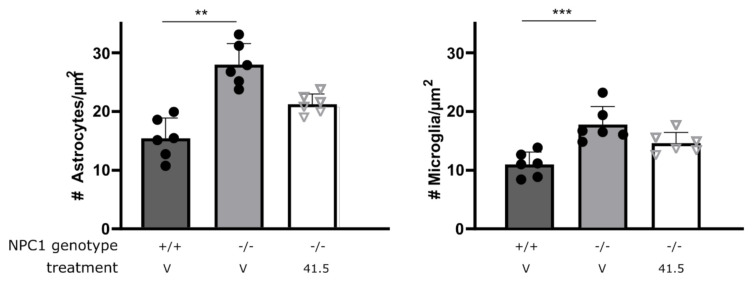
MSC41.5-EV treatment of NPC1^−/−^ mice reduces inflammation in different brain regions. NPC1^−/−^ mice and wild type littermates are treated four times by intravenous injection into lateral tail. At age of 7 weeks, brain was isolated and IBA and GFAP staining was performed on hippocampus. Data are shown as mean ± SEM (*n* = 6 mice per group). Statistical analyses on datasets were performed by Kruskal–Wallis test. Asterisks indicate statistical significance (** *p* < 0.01, *** *p* > 0.001).

Astrocytosis and microgliosis are typical features of NPC1 pathology [6,43,44]. Analysis of astrocyte and microglia numbers in the hippocampus of NPC1^−/−^ mice via IBA1 and GFAP stainings, respectively, revealed astrocytosis and microgliosis due to NPC1 deficiency (Figure 4). Again, this phenotype could almost completely be mitigated by the administration of MSC41.5-EVs in NPC1^−/−^ mice.

## 3. Discussion

Niemann–Pick disease type C1 (NPC1) is a rare progressive genetic disorder characterized by an inability of cells to transport cholesterol and lipids. This results in the abnormal accumulation of these substances within various tissues of the body, leading to a broad spectrum of visceral and neurological symptoms. Up to now, only symptomatic medication is available that alters specific NPC1-associated symptoms while no cure exists. As different studies show the immunomodulatory effect of EVs harvested from cell culture supernatants of humans MSCs [21,26,45,46,47], we here intravenously administered MSC-EVs harvested from different healthy human MSC donors (sources 16.3 and 41.5) in a mouse model for NPC1 pathology (NPC1^−/−^) to investigate the modulatory effects on typical NPC1 disease features.

Infants with NPC1 usually develop splenomegaly by the age of 3 months and fail to gain weight and grow at the expected rate. Next to this, the inflammatory cytokine tumor necrosis factor (TNF) was suggested as an important cytokine in NPC1 disease progression as multiple members of the TNF pathway are overexpressed in peripheral organs of NPC1^−/−^ mice [40,41,48]. This pro-inflammatory cytokine plays an important role in apoptosis, inflammation and fibrosis, typical features of NPC1 diseased peripheral organs [36,38]. For example, it is shown that in the liver TNF that is secreted by foamy macrophages attract inflammatory cells and stimulate hepatic stellate cells, which deposit collagen and signals apoptosis of hepatocytes [34,35,37,39]. Moreover, Rimkunas et al. showed that by utilizing NPC1-specific antisense oligonucleotides to knock down NPC1 expression in control and TNF knockout mice, in the absence of TNF inflammation, apoptosis and fibrosis were present [40].

Here, we show that treatment with EVs isolated from MSCs of donor 41.5 is able to alter the above-described typical visceral features of NPC1 pathology. More precisely, we observed that treatment of NPC1 deficient mice with MSC-EV type 41.5 improves weight gain and reduces spleen enlargement. The treatment also reverses the increased expression of the inflammatory cytokine *Tnf* in the liver and the lungs and remarkably decreases the amount of foam cells in the liver, spleen, and lungs.

Next to the pronounced effects on the peripheral pathology, also a clear improvement of the neurological symptoms was observed after treatment of NPC1^−/−^ mice with MSC-EV 41.5. Indeed, neuroinflammation, determined via gene expression analysis of *Ccl3*, *Ccl5*, *Cxcl10*, and *Tnf*, was almost completely reversed upon treatment with MSC41.5-EVs in all brain regions tested. Even more striking is the observation that MSC-EV 41.5 is able to reduce the level of astrogliosis and microgliosis, two prominent neuropathological features of the NPC1 disease. A possible explanation, however speculative, for the weight gain in NPC1^−/−^ mice upon treatment with MSC-EV type 41.5 is that a reduced severity of the peripheral and neurological symptoms, e.g., systemic inflammation and neuroinflammation results in less overall sickness behavior, which in turn leads to an increased/better appetite with the NPC1 diseased mice.

All these findings underline the therapeutic potential of EVs derived from MSC as treatment strategy for the genetic neurovisceral lipid storage disease NPC. Moreover, an EV-based therapy has various advantages over cell therapies as they are not self-replicating and as they can hardly sense environmental conditions, their biological activity can be predicted more precisely than that of cells [23]. However, important to keep in mind is that huge differences among independent MSC-EV preparations are reported, e.g., in cytokine profiles [11,26]. Recently, Madel et al. showed therapeutic differences among independent MSC-EV preparations that had been produced in a standardized manner [32]. Also in our study, we show that the effects observed upon treatment with MSV-EV type 41.5 are in shear contrast to the results obtained upon treatment with EVs isolated from MSC of donor 16.3. Indeed, despite treatment with similar particle numbers of EVs isolated from donor 16.3 MSCs compared to 41.5, the investigated features were not reduced and were comparable to NPC1^−/−^ mice treated with vehicle or platelet derived EVs. These results are in agreement with the in vitro immunomodulatory capacity of both MSC-EV fractions [32,33]. Consequently, functional testing of any MSC-EV preparation is necessary prior to clinical application. Unfortunately, it is currently nondeducible from the MSCs phenotypes whether resulting EV preparations have a higher or lower chance to be therapeutically active. So, before MSC-EV treatment strategies can be used to treat NPC1 patients, reliable methodologies need to be developed to determine the in vivo immunomodulatory potential of a specific MSV-EV batch.

Taken together, MSC-EV administration modulates NPC1 pathology in mice on the level of visceral as well as neurologic symptoms. These results may open new treatment perspectives for the so far hard to treat disease NPC1.

## 4. Materials and Methods

### 4.1. Mice

Balb/cNctr-*Npc1^m1N^*/J mice aged five to seven weeks were used. These mice harbor an insertion in chromosome 18, resulting in a premature truncation of the NPC1 protein deleting 11 out of 13 transmembrane domains. In all experiments, mice were age- and gender-matched with wild type littermates. All mice were housed with 14 to 10 h light and dark cycles and free access to food and water in SPF conditions. All experiments comply with the current laws of Belgium and were approved by the animal ethics committee of Ghent University (EC 2015-052; 29/10/2015).

### 4.2. Preparation of EVs

MSCs were raised from bone marrow samples of two healthy human donors exactly as described previously [26,49]. For the EV harvesting, MSCs from two different MSC stocks, MSC16.3 and MSC41.5, were expanded at 37 °C in a 5% CO_2_ atmosphere in DMEM low glucose basal media (PAN Biotech, Aidenbach, Germany), supplemented with 10% human platelet lysate, 100 U/mL penicillin-streptomycin-glutamine (Thermo Fisher Scientific, Darmstadt, Germany) and 5 IU/mL Heparin (Ratiopharm, Ulm, Germany) exactly as described previously [26]. Starting at ~50–80% confluency, media exchanges were performed every 48 h. For the cryopreservation at −20 °C, larger debris were removed from the conditioned media by 2000× *g* centrifugation for 15 min (Rotor: JS-5.3; Beckman Coulter, Brea, CA, USA). After thawing, MSC-EVs as well as platelet EVs from non-conditioned, fresh MSC-media were prepared using an optimized polyethylene glycole 6000 precipitation protocol followed by ultracentrifugation exactly as reported previously [26,31]. The EV harvest of conditioned media of 4 × 10^7^ MSCs were solved in 1 mL 10 mM HEPES (Gibco, Waltham, MA, USA) 0.9% NaCl (bBraun, Bucharest, Romania) buffer and stored at −80 °C until usage. Both MSC-EV and the PL EV preparation were characterized according to the minimal information for studies of extracellular vesicles 2018 (MISEV2018) guidelines [50]. The same MSC-EVs are used as by Wang et al. and Madel et al. [32,33]. Full characterization of the MSC-EV fractions was reported previously in [33]. Table 1 gives an overview of the protein concentration and size of the used EVs (Table 1).

### 4.3. Treatment

The mice were intravenously injected with 4 × 10^4^ cell equivalents (CE) EVs/g diluted in 10 mM HEPES in 0.9% NaCl buffer or with vehicle only as negative control. The treatment was administered four times over a period of two weeks, starting when the mice were 5 weeks of age. At the age of 7 weeks, the mice were sacrificed (Figure 1A). The study was performed blinded, meaning that the activity of the MSC-EV preparations was unknown for the researchers performing the injections and evaluating the effect of the treatment on NPC1 pathology.

### 4.4. Tissue Isolation

For RNA or protein analysis, mice were transcardially perfused with a D-PBS/heparin (0.2% heparin (5000 IU/mL, Wockhardt, Mumbai, India)) supplemented with 0.5% bromophenol blue (Sigma, St. Louis, MO, USA). Spleen, liver, lung, hippocampus, cerebellum, olfactory bulb, prefrontal cortex was dissected out and snap-frozen in liquid nitrogen. For immunohistochemical analysis, mice were transcardially perfused with 4% PFA, and spleen, liver, lung, and brain samples were isolated, fixed in 4% PFA and processed for paraffin-embedding.

### 4.5. RNA Isolation and Real-Time qPCR

RNA was isolated from all tissues using the Aurum total RNA Mini Kit (Bio-Rad), according to the manufacturer’s instructions. RNA concentration and purity were determined spectrophotometrically using the Nanodrop ND-1000 (Nanodrop Technologies, Thermo Scientific, Bucharest, Romania) and cDNA was synthesized with the SensiFAST™ cDNA Synthesis Kit (Bioline, London, UK). RT-qPCR was performed with the Light Cycler 480 system (Roche, Basel, Switzerland) using SensiFast SYBR No-Rox (Bio-Line). Expression levels were normalized to the expression of two or three most stable reference genes, determined using the geNorm Housekeeping Gene Selection Software. The sequences of the forward and reversed primers for the different genes are provided (Table 2).

### 4.6. Immunohistochemistry

For immunostaining on mouse brain sections, 5 µm sections were prepared from paraffin-embedded samples. Sections were dewaxed using Varistain (Thermo Fisher Scientific), followed by an antigen retrieval step using citrate buffer (Vector; H-3300) and wash steps with PBS-T (PBS supplemented with 0.5% Triton X-100). Next, endogenous peroxidase activity was blocked with 3% H_2_O_2_ in methanol for 10 min. Samples were blocked with bovine serum albumin (BSA) and 5% normal goat serum in PBS-T (0.5% (*w*/*v*) BSA and 0.02% (*v*/*v*) Triton X-100 in PBS) for 30 min at RT, followed by overnight incubation at 4 °C with primary antibody against GFAP (1:1000; Agilent; Z033429-2) or IBA1 (1:200; Wako Chemicals, 019-19741). After ON incubation, slides were washed with PBS-T and incubated with goat anti-rabbit biotinylated antibody (1:500; Dako, E0432). Visualization was done using ABC (Vector; PK6100) and DAB. Next, slides were counterstained with hematoxylin, dehydrated to xylene and mounted with Entellan. Image acquisition was performed using a slide scanner (Zeiss, Axio Scan) and analyzed using the Zen software (Carl Zeiss Microscopy GmbH, Jena, Germany, 2012).

## 5. Statistical Analysis

Statistical analyses were performed using GraphPad Prism 9.0 (GraphPad Software, Inc. San Diego, CA, USA). Bars represent mean ± SEM. qPCR data were analyzed with an unpaired *t*-test unless mentioned differently. Significance levels are indicated * 0.01 ≤ *p* < 0.05; ** 0.001 ≤ *p* < 0.01; *** 0.001 ≤ *p* < 0.0001; and **** *p* < 0.0001.

## Figures and Tables

**Table 1:** Overview of protein concentration and size (NTA-determined) of used EVs.

EV Type	Protein Concentration (µg/µL)	Size (nm)
MSC41.5-EVs	4.80	125.6
MSC16.3-EVs	4.89	114.5
PL EV	7.52	125.2

**Table 2:** Overview of used RT-qPCR primer sequences.

Gene	Forward	Reverse
***Gapdh***	TGAAGCAGGCATCTGAGGG	CGAAGGTGGAAGAGTGGGAG
***Hprt***	AGTGTTGGATACAGGCCAGAC	CGTGATTCAAATCCCTGAAGT
***Rpl***	CCTGCTGCTCTCAAGGTT	TGGTTGTCACTGCCTCGTACTT
***Ubc***	AGGTCAAACAGGAAGACAGACGTA	TCACACCCAAGAACAAGCACA
***Ccl3***	TTCTCTGTACCATGACACTCTGC	CGTGGAATCTTCCGGCTGTAG
***Ccl5***	GCTGCTTTGCCTACCTCTCC	TCGAGTGACAAACACGACTGC
***Cxcl10***	CCAAGTGCTGCCGTCATTTTC	GGCTCGCAGGGATGATTTCAA
***Tnf***	ACCCTGGTATGAGCCCATATAC	ACACCCATTCCCTTCACAGAG

## Data Availability

The original contributors presented in the study are included in the article/supplementary Material, further inquiries can be directed to the corresponding author.

## Supplementary Materials

The following are available online at https://www.mdpi.com/article/10.3390/biomedicines9121864/s1, Figure S1: NPC1^−/−^ mice show a progressive lower weight compared to their wildtype littermates, Figure S2: MSC-EV type 16.3 has no effect on NPC1^−/−^ pathology in peripheral organs, Figure S3: MSC-EV 16.3 treatment of NPC1^−/−^ mice has no effect on inflammation in different brain regions
