# Supplementary material for: Anti-Inflammatory Mesenchymal Stromal Cell-Derived Extracellular Vesicles Improve Pathology in Niemann–Pick Type C Disease
Source: Biomedicines. 2021 Dec 8;9(12):1864. doi: 10.3390/biomedicines9121864 (PMC8698931; doi:10.3390/biomedicines9121864)
Supplement: Supplementary file 1 [file biomedicines-09-01864-s001.zip › biomedicines-1444442-supplementary.pdf]

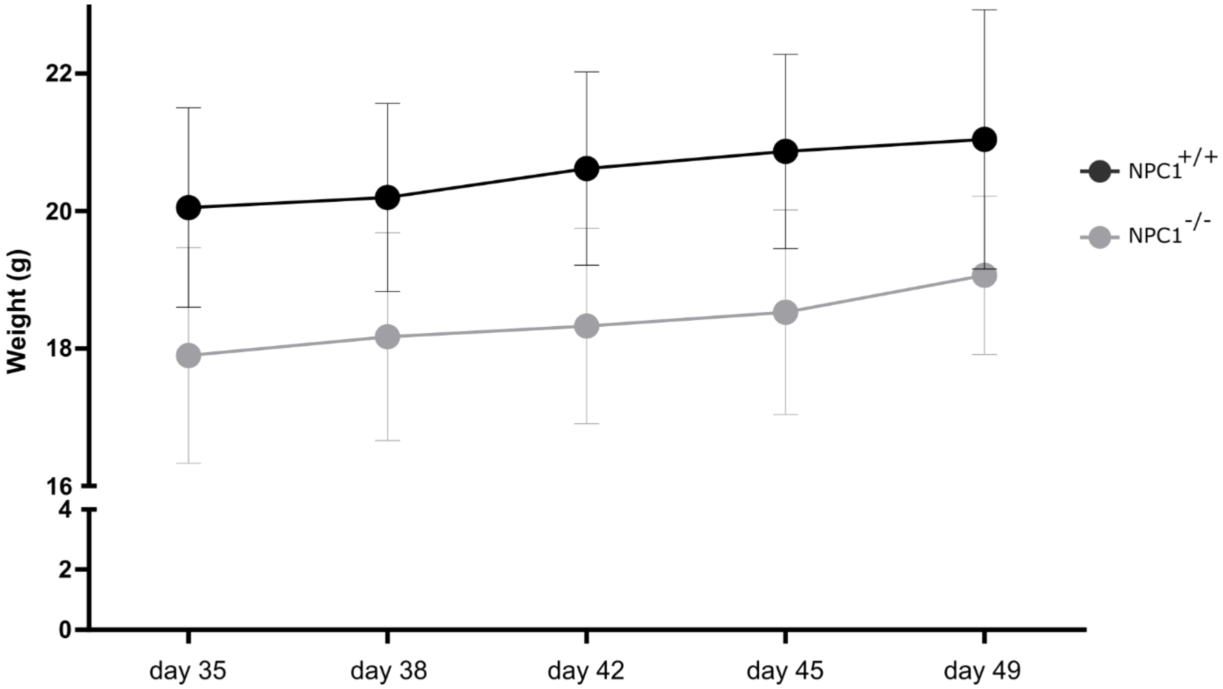

Figure S1: NPC1$^{-/-}$ mice show a progressive lower weight compared to their wildtype littermates. Changes in mean body weight of NPC1$^{-/-}$ and NPC1$^{+/+}$ mice from day 35 to 49 after birth. Data are shown as mean ± SEM (n=10 mice per group).

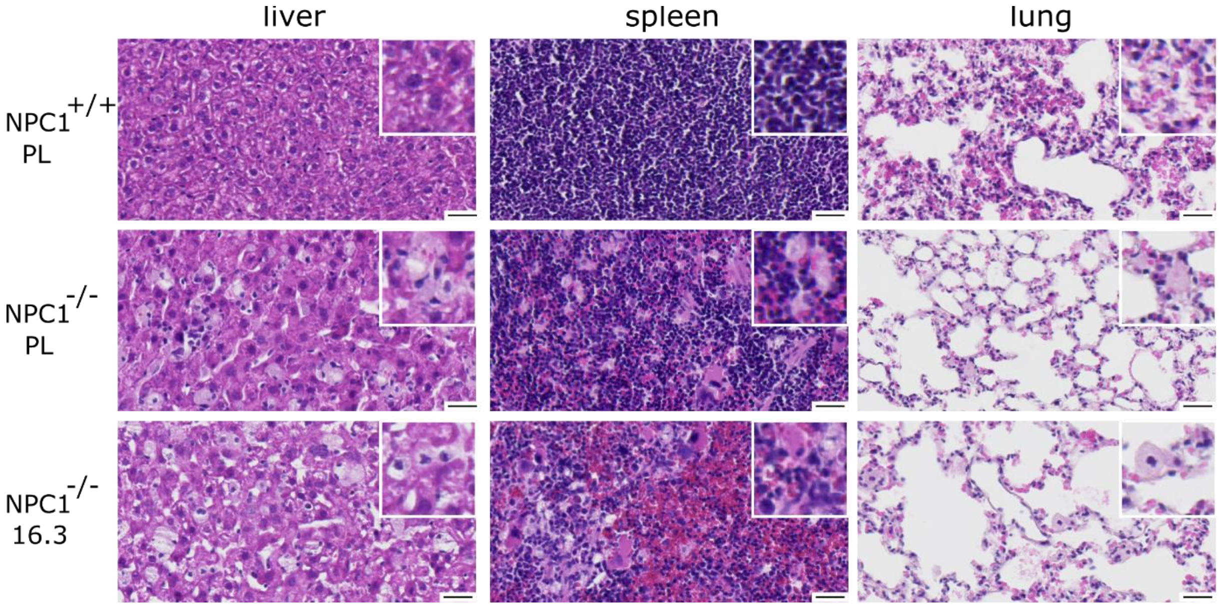

Figure S2: MSC-EV type 16.3 has no effect on NPC1$^{-/-}$ pathology in peripheral organs. NPC1$^{-/-}$ mice and wild type littermates are in the course of two weeks four times injected intravenous into the lateral tail vein with vehicle, platelet derived EVs (PL EVs) or MSC-EVs derived from human MSC donor 16.3 (MSC16.3-EVs). At the age of 7 weeks, liver, spleen and lungs were isolated and hematoxylin and eosin (H&E) staining was performed on these organs. PL: platelet derived EVs; 16.3: MSC16.3-EVs. The scale bars represent 50 µm.

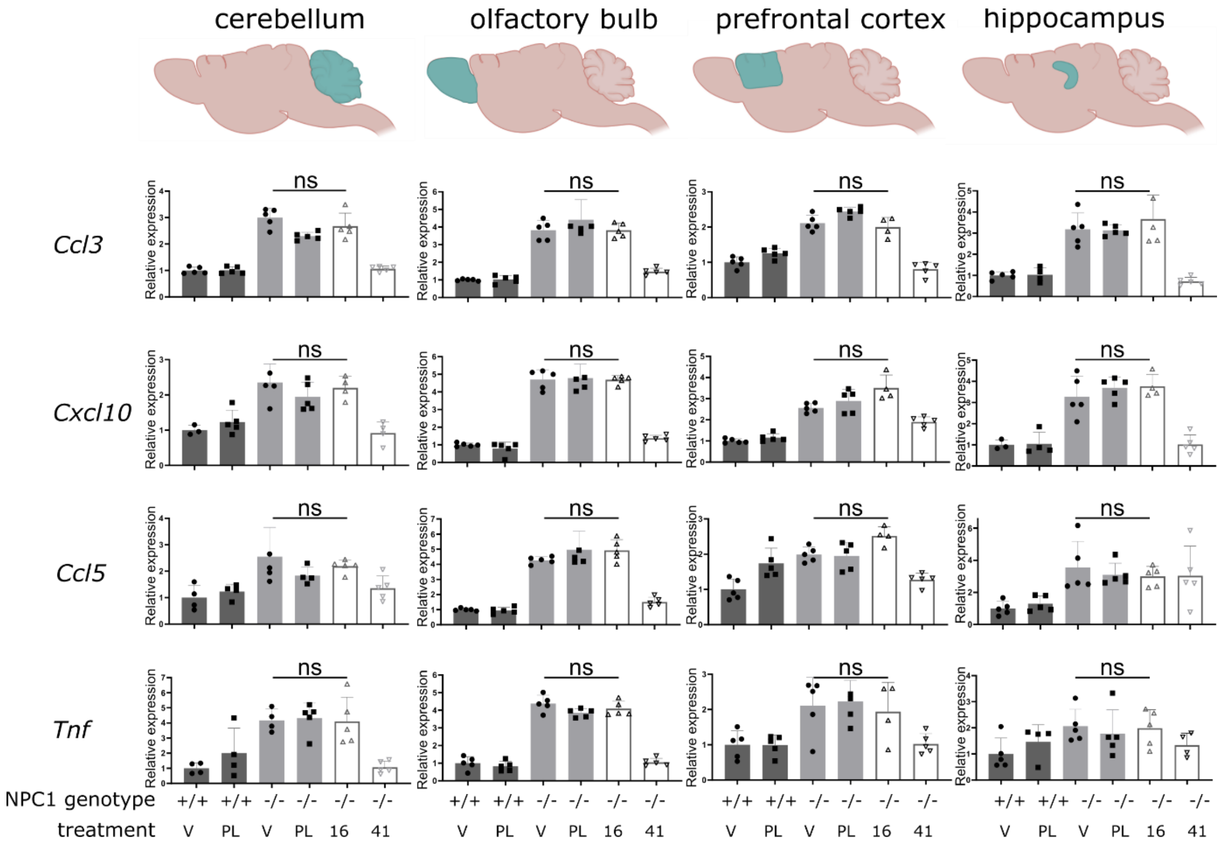

Figure S3: MSC-EV 16.3 treatment of NPC1$^{-/-}$ mice has no effect on inflammation in different brain regions. NPC1$^{-/-}$ mice and wild type littermates are four times injected intravenous into the lateral tail vein with vehicle, platelet derived EVs (PL EVs), MSC-EVs derived from human MSC donor 16.3 (MSC-EV 16.3) or MSC-EVs derived from human MSC donor 41.2 (MSC-EV 41.2). At the age of 7 weeks, different brain regions were isolated and *Ccl3*, *Cxcl10*, *Ccl5* and *Tnf* gene expression was analyzed. Results are represented relative to the NPC1$^{+/+}$ vehicle condition. Data are shown as mean ± SEM (n=5 mice per group). Statistical analyses on datasets were performed by Mann-Whitney test. ns: non significant).
